# Transplantation of Rabbits' Conjunctiva to the Human Eye

**Published:** 1890-02-15

**Authors:** 


### SURGERY.

### TRANSPLANTATION OF RABBITS' CONJUNCTIVA TO THE HUMAN EYE.

Dr. J. R. Wolfe, Professor of Ophthalmology in St. Mungo's College, has written for the *Lancet* a paper "On Transplantation of Rabbits' Conjunctiva to the Human Eye," with illustrations. After detailing the reasons which led to the adoption of transplantation, Dr. Wolfe gives a case. A steel-burner got his eye injured by a flash of slag nine years ago. Attempts were made to keep the eyelids apart, but notwithstanding, they became completely closed, the free borders of the lids being obliterated, so that dissection of the upper and lower lids from the globe was effected with difficulty. It was then found that only the upper and outer quadrant of the cornea was transparent. The lids were kept apart with ligatures, and an artificial pupil formed in front of the transparent cornea. The symblepharon was subsequently remedied by transplanting conjunctiva from the rabbit. The eye is now moveable in every direction, and vision is restored.

